# The position of the aorta relative to the spine in patients with adult degenerative scoliosis

**DOI:** 10.1186/s13018-020-1578-y

**Published:** 2020-02-24

**Authors:** Yan Liang, Shuai Xu, Yongfei Zhao, Zhenqi Zhu, Keya Mao, Zheng Wang, Haiying Liu

**Affiliations:** 1 Department of Spinal Surgery, Peking University People’s Hospital, No. 11 Xi Zhimen South Street, Xi Cheng District, Beijing, 100044 China; 2 Orthopedic Department, The Chinese PLA General Hospital (301 Hospital), No. 28 Fu Xing Rd, Hai Dian District, Beijing, 100853 China

**Keywords:** Adult degenerative scoliosis, Aorta, Aorta-vertebrae angle, Aorta-vertebrae distance, Rotation angle

## Abstract

**Study design:**

A retrospective analysis was conducted to analyze the position of the aorta by MRI in patients with adult degenerative scoliosis.

**Objective:**

This study aimed to investigate the relative anatomic positions of the aorta and spine in patients with adult degenerative scoliosis (ADS).

**Summary of background data:**

Aorta injury is a rare complication of spinal surgeries. However, there would be a disastrous consequence once it happened. Therefore, knowing about the position of aorta is of great importance.

**Methods:**

A retrospective analysis was performed in 90 patients with ADS and 132 participants without spine deformity. ADS patients were divided into several groups such as left scoliosis, left scoliosis with thoracolumbar kyphosis, right scoliosis, and right scoliosis with thoracolumbar kyphosis. The aorta-vertebrae angle (*α*) and aorta-vertebrae distance (*d*) in each level of T12–L4 were measured by using a Cartesian coordinate system. *t* test of independent samples was performed, *α* and *d* were compared, and Pearson correlation analysis was employed for *α*, *d*, and X-ray radiographic measurements.

**Result:**

The changes of *α* were not statistically significant (*P* > 0.05) in LS and LKS groups but *d* (*P* < 0.05) was longer in LKS group compared with the control group. In the right malformed group, there was no significant change in the angle (*P* > 0.05) in the abdominal aorta but longer *d* (*P* < 0.05) than the normal group. There was longer *d* in the RKS group compared with the RS group (*P* < 0.05). Pearson correlation analysis showed that there was a positive correlation between *d* and TLK (*r* = 0.439, *P* < 0.05).

**Conclusion:**

In patients with ADS, a relative normal position is maintained between the aorta and vertebrae. While the aorta is slightly away from the left pedicle in RS patients and farther away in patients with kyphosis, the angle of kyphosis would become bigger and *d* becomes longer. Therefore, the surgeons should be aware of the changes of the aorta position to avoid the disastrous vessel injuries.

## Introduction

ADS (adult degenerative scoliosis) was defined as a curve > 10° due to asymmetrical degeneration of the facets and discs which apply an asymmetric load on the spine leading to degenerative scoliosis [1–3]. In ADS, there is a major curve on the lumbar spine complicated by lateral and rotatory subluxation. Therefore, deformity correction surgery was necessary and osteotomy was required to achieve a good balance. Due to the complexity in the posterior tissue, the insertion of pedicle screws appeared to be difficult and risky. Besides, ADS patients were usually accompanied by advanced age and atherosclerosis; thus, the vascular elasticity was reduced. All these factors could lead to aorta injury during the surgeries. Although aorta injury is rare among the complications, the consequence would be disastrous once it occurred. Previous studies reported that the manifestation of aorta injury is mainly bleeding and pseudoaneurysm [4, 5]. Several studies have evaluated the relative position of the aorta and the spine in adolescent patients with idiopathic scoliosis and kyphosis deformity [6–10]. Nonetheless, there was no study investigating the relative anatomic position of aorta and spine in patients with ADS. Therefore, our research was conducted to explore the relative anatomic position of the aorta and spine in patients with ADS.

## Materials and methods

### Participants

This retrospective single-center study was approved by the medical ethic committee of our hospital. A total of 132 subjects without spine deformity (control group) and 90 patients with DLS in our hospital were recruited from January 2014 to June 2018. Twenty-six patients with DLS were assigned into the left lumbar scoliosis group (LS group), 30 patients into the left kyphoscoliosis group (LKS group), 20 patients into the right scoliosis group (RS group), and 14 patients into the right kyphoscoliosis group (RKS group). Scoliosis was defined as no sagittal local malformation while LKS or RKS was defined as a thoracolumbar kyphosis (TLK) > 15° without any other deformities.

The inclusion criteria are as follows: (1) the apical vertebrae were located within thoracolumbar or lumbar spine (T12–L4), (2) magnetic resonance images (MRI) of the thoracolumbar and lumbosacral spines were available, and (3) posteroanterior and lateral radiographs containing lumbar and the whole spine were available. The exclusion criteria are follows: (1) congenital vascular abnormalities, (2) previous spinal surgeries, or (3) previous cardiovascular surgeries. Informed consents were obtained from all subjects.

There was no significant difference in sex distribution among the LS, LKS, and control groups (*P* = 0.775) and between the RS and RKS groups (*P* = 0.394). The age (*P* = 0.109 and *P* = 0.785, respectively) and the body mass index (BMI) (*P* = 0.555 and *P* = 0.058, respectively) were well-matched in the LS and LKS groups and RS and RKS groups compared with the control group (Table 1).

**Table 1:** Demographic characteristics of deformity and control participants

	Control	LS	LKS	*P*	Control	RS	RKS	*P*
Sex				0.775				0.394
Male	20	2	4		20	6	4	
Female	112	24	26		112	14	10	
Age, year	63.30 ± 8.42	66.08 ± 7.18	67.87 ± 7.08	0.109	63.30 ± 8.42	65.90 ± 6.47	63.43 ± 8.83	0.785
Height, m	1.61 ± 0.06	1.59 ± 0.05	1.60 ± 0.07	0.567	1.61 ± 0.06	1.62 ± 0.08	1.63 ± 0.09	0.549
Weight, kg	67.85 ± 10.36	72.81 ± 9.92	68.93 ± 9.09	0.275	67.85 ± 10.36	61.30 ± 7.79	72.57 ± 13.95	0.078
BMI, kg/m^2^	26.29 ± 3.66	28.89 ± 3.40	26.80 ± 2.76	0.555	26.29 ± 3.66	23.46 ± 2.29	27.20 ± 5.29	0.058

*LS* left scoliosis group, *LKS* left kyphoscoliosis group, *RS* right scoliosis group, *RKS* right kyphoscoliosis group, *BMI* body mass index

### Measurements

#### Measurements on X-ray radiograph

The posterior-anterior and lateral X-ray films of the lumbar and whole spine were obtained in standard standing position to identify (1) LS, LKS, RS, or RKS scoliosis; (2) coronal Cobb angle (°); (3) apical vertebrae distribution; (4) coronal horizontal displacement distance (mm) (the vertical distance from the curvature apex to the sacral vertical line); and (5) TLK (the sagittal angle between superior endplate of T10 and inferior endplate of L2, which was a positive value in kyphosis patients). The parameters were obtained by two independent investigators.

#### Measurements on MRI

All subjects were required to lie in a neutral supine position. MRI was obtained using a 1.5-T scanner (Gyroscan Intera; Philips Medical Systems, NL). Axial slices (4 mm) with 1-mm overlap were acquired using a three-dimensional thick T2-weighted spin-echo axial scan through the vertebral bodies (TR, 5000 ms; TE, 120 ms; FOV, 250 mm; matrix size, 250 × 360). The same MR scans and image acquisition protocol were applied for supine position. Images were analyzed using PACS client software (Easy Vision IDS5, version 11.4; Philips, Hamburg, Germany). To clarify the relative positions of the abdominal aorta and the vertebrae, the following parameters were measured in the MR images from the T12 vertebrae to the L5 vertebrae with a Cartesian coordinate system [11].

##### Cartesian coordinate system

A line connecting both medial edges of the superior facets was defined as the *x*-axis. The *y*-axis was perpendicular to the *x*-axis starting from the dorsal edge of the right superior facet. The two axes intersect at the origin O.

##### Left pedicle-aorta angle (*α*)

The left pedicle-aorta angle (*α*) was formed by the *y*-axis and a line connecting the origin and the center of the aorta. The angle was 90° when the aorta was located on the left side and − 90° when it was on the right side of the origin.

##### Left pedicle-aorta distance (*d*)

**Fig. 1:**
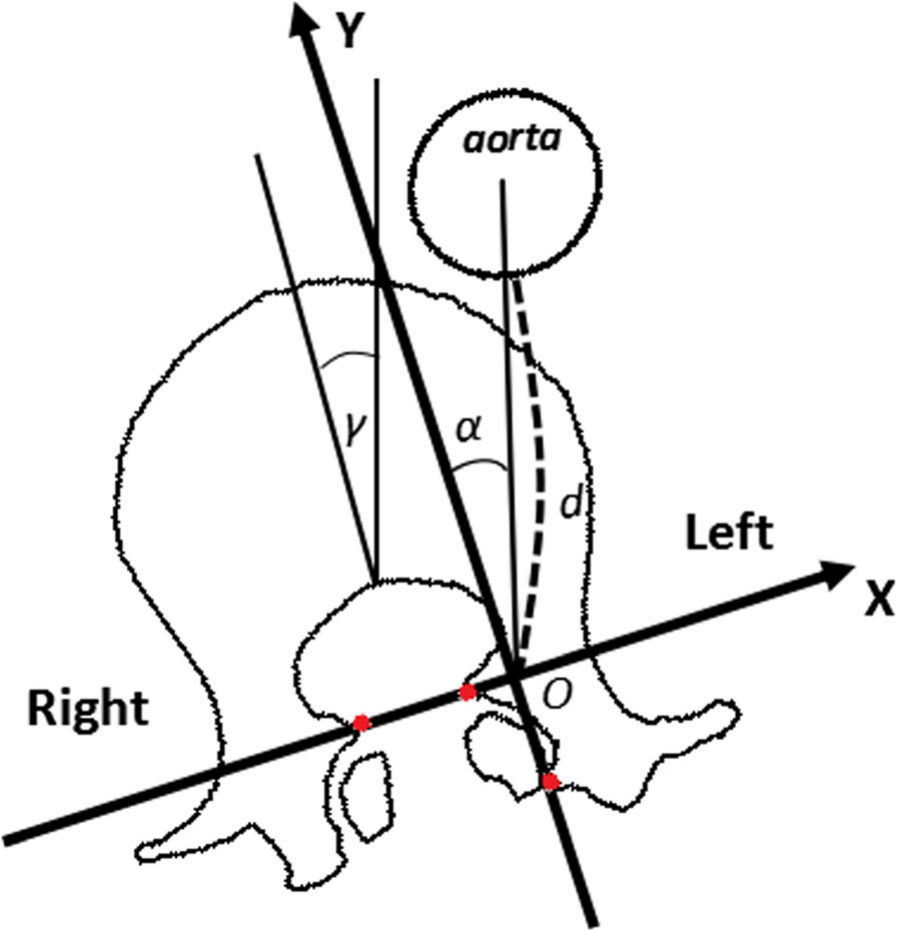
The position of Cartesian coordinate system and instructions for *α*, *γ*, and *d*

This distance was defined as a line connecting the origin O and the nearest edge of the aorta (Fig. 1).

### Statistical analysis

The values of each parameter at each vertebral level were presented as mean ± standard deviation. Independent samples *t* test was performed to compare *α*, *γ*, and *d* respectively between the four DLS groups and control group, between the LS and LKS groups, and between the RS and RKS groups. Pearson correlation analysis was employed for Cobb angle, the horizontal displacement distance, and *α*, *γ*, and *d* in the four deformity groups. The data were analyzed using SPSS 22.0 software. If *P* < 0.05, the data were considered significantly different.

## Results

### Measurements on X-ray radiograph

In the whole left group, L1 to L4 was distributed as apical vertebrae, of which L3 (53.8%) was the most in LS group while L2 (40.0%) and L3 (33.3%) were the majority in LKS group. There was no statistical difference in apical vertebrae distribution between the LS and LKS groups. Moreover, there was no significant difference in the average Cobb angle and coronal horizontal displacement distance between the LS and LKS groups (*P* = 0.088 and *P* = 0.195, respectively). Apical vertebrae could be identified from T12 to L4 which were mainly located on L2 (50.0%) in the RS group and L3 in the RKS group. However, there was no difference between the two groups in apical vertebrae distribution (*P* = 0.163). The average Cobb angle and coronal horizontal displacement distance were also of no statistical difference between the RS and RKS groups (*P* = 0.743 and *P* = 0.353, respectively) (Table 2).

**Table 2:** Cobb angle, apical vertebrae distribution, and coronal horizontal displacement distance in the four malformed groups

	Ap-V in LS	Ap-V in LKS	*P*	Ap-V in RS	Ap-V in RKS	*P*
T12	0	0	0.301	1	0	0.163
L1	2	2		2	1	
L2	1	6		5	2	
L3	7	5		2	4	
L4	3	2		0	0	
Cobb angle, °	19.78 ± 7.86	28.44 ± 16.74	0.088	22.36 ± 10.28	24.20 ± 12.46	0.743
Coronal movement, mm	42.21 ± 9.79	47.90 ± 12.41	0.195	52.18 ± 12.00	58.02 ± 12.95	0.353
TLK, °	10.26 ± 8.37	19.50 ± 12.12	0.001	11.85 ± 11.72	33.53 ± 18.89	< 0.001

*Ap-V* apical vertebrae distribution, *LS* left scoliosis group, *LKS* left kyphoscoliosis group, *RS* right scoliosis group, *RKS* right kyphoscoliosis group, *TLK* thoracolumbar kyphosis

### Measurements on MRI

#### Comparisons of *α* and *d* among LS, LKS, and control groups

**Fig. 2:**
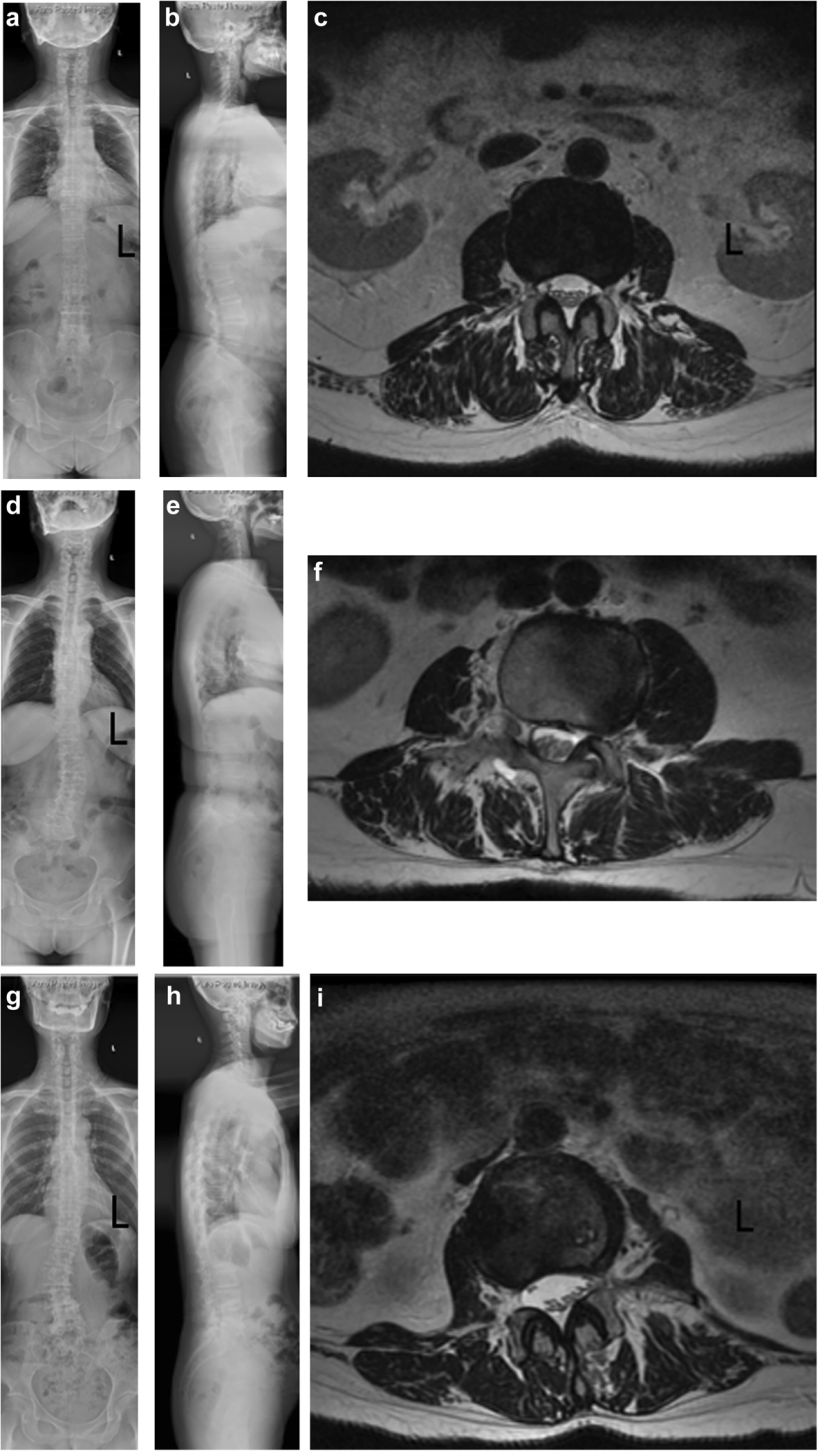
The standard standing whole spine posterior-anterior and lateral X-ray and lumbar spine MRI T2-weighted axis image of the control, LS, and LKS groups. **a–c** The whole spine X-ray and L3 level MRI of 63-year-old women in the control group with *α* of − 4.1° and *d* of 4.60 cm. **d–f** The whole spine X-ray and L3 level MRI of 61-year-old women in the LS group. The TLK is 3.5° with no defined kyphosis. The apical vertebrae is L3 and Cobb angle is 21.3° with *α* of −  4.8°, *γ* of 6.3°, and *d* of 5.12 cm. **g–i** The whole spine X-ray and L3 level MRI of 67-year-old women in the LKS group. The TLK is 34.3° with kyphosis. The apical vertebrae is L3 and Cobb angle is 16.7° with *α* of 5.9°, *γ* of 15.6°, and *d* of 5.43 cm

There were no significant differences in *α* between the LS (− 5.27 ± 7.15°) and control groups (− 3.56 ± 6.40°) (*P* = 0.063), LKS (− 3.43 ± 7.75°) and control groups (*P* = 0.876), or LS and LKS groups (*P* = 0.107). In the control group, *d* (4.45 ± 0.43 cm) gradually increased from T12 to L4, indicating that abdominal aorta moved ventrally away from the left vertebral pedicel along the descending trace, and similar results could be obtained in the LS group (4.51 ± 0.49 cm) and LKS group (4.70 ± 0.79 cm). There were significant differences between the LKS and control groups (*P* < 0.001) or LS and LKS groups (*P* = 0.035), but no significant differences between the LS and control groups (*P* = 0.327) (Table 3) (Fig. 2).

**Table 3:** Comparisons on *α* and *d* from T12 to L4 among LS, LKS, and control groups

		LS vs control		LKS vs control		LS vs LKS	
*α*, °	T12	− 1.96 ± 10.06	− 1.94 ± 7.02	− 2.90 ± 10.04	− 1.94 ± 7.02	− 1.96 ± 10.06	− 2.90 ± 10.04
	L1	− 3.93 ± 6.12*	0.30 ± 5.20	− 3.39 ± 7.51*	0.30 ± 5.20	− 3.93 ± 6.12	− 3.39 ± 7.51
	L2	− 7.30 ± 4.06**	− 3.17 ± 4.81	− 3.39 ± 5.14	− 3.17 ± 4.81	− 7.30 ± 4.06	− 3.39 ± 5.14*
	L3	− 3.69 ± 7.67	− 5.41 ± 5.82	− 1.65 ± 8.99	− 5.41 ± 5.82	− 3.69 ± 7.67	− 1.65 ± 8.99
	L4	− 9.48 ± 4.35	− 7.63 ± 6.13	− 5.79 ± 6.56	− 7.63 ± 6.13	− 9.48 ± 4.35	− 5.79 ± 6.56
	Mean	− 5.27 ± 7.15	− 3.56 ± 6.40	− 3.43 ± 7.75	− 3.56 ± 6.40	− 5.27 ± 7.15	− 3.43 ± 7.75
*d*, cm	T12	4.03 ± 0.58	4.02 ± 0.39	3.89 ± 1.24	4.02 ± 0.39	4.03 ± 0.58	3.89 ± 1.24
	L1	4.33 ± 0.42	4.26 ± 0.29	4.58 ± 0.45**	4.26 ± 0.29	4.33 ± 0.42	4.58 ± 0.45*
	L2	4.61 ± 0.31	4.50 ± 0.30	4.91 ± 0.36**	4.50 ± 0.30	4.61 ± 0.31	4.91 ± 0.36*
	L3	4.76 ± 0.29	4.70 ± 0.34	5.05 ± 0.50**	4.70 ± 0.34	4.76 ± 0.29	5.05 ± 0.50*
	L4	4.85 ± 0.33	4.71 ± 0.38	5.05 ± 0.36**	4.71 ± 0.38	4.85 ± 0.33	5.05 ± 0.36
	Mean	4.51 ± 0.49	4.45 ± 0.43	4.70 ± 0.79**	4.45 ± 0.43	4.51 ± 0.49	4.70 ± 0.79*

*LS* left scoliosis group, *LKS* left kyphoscoliosis group

*Significance with *P* < 0.05

**Significance with *P* < 0.01

#### Comparisons of *α* and *d* among RS, RKS, and control groups

**Fig. 3:**
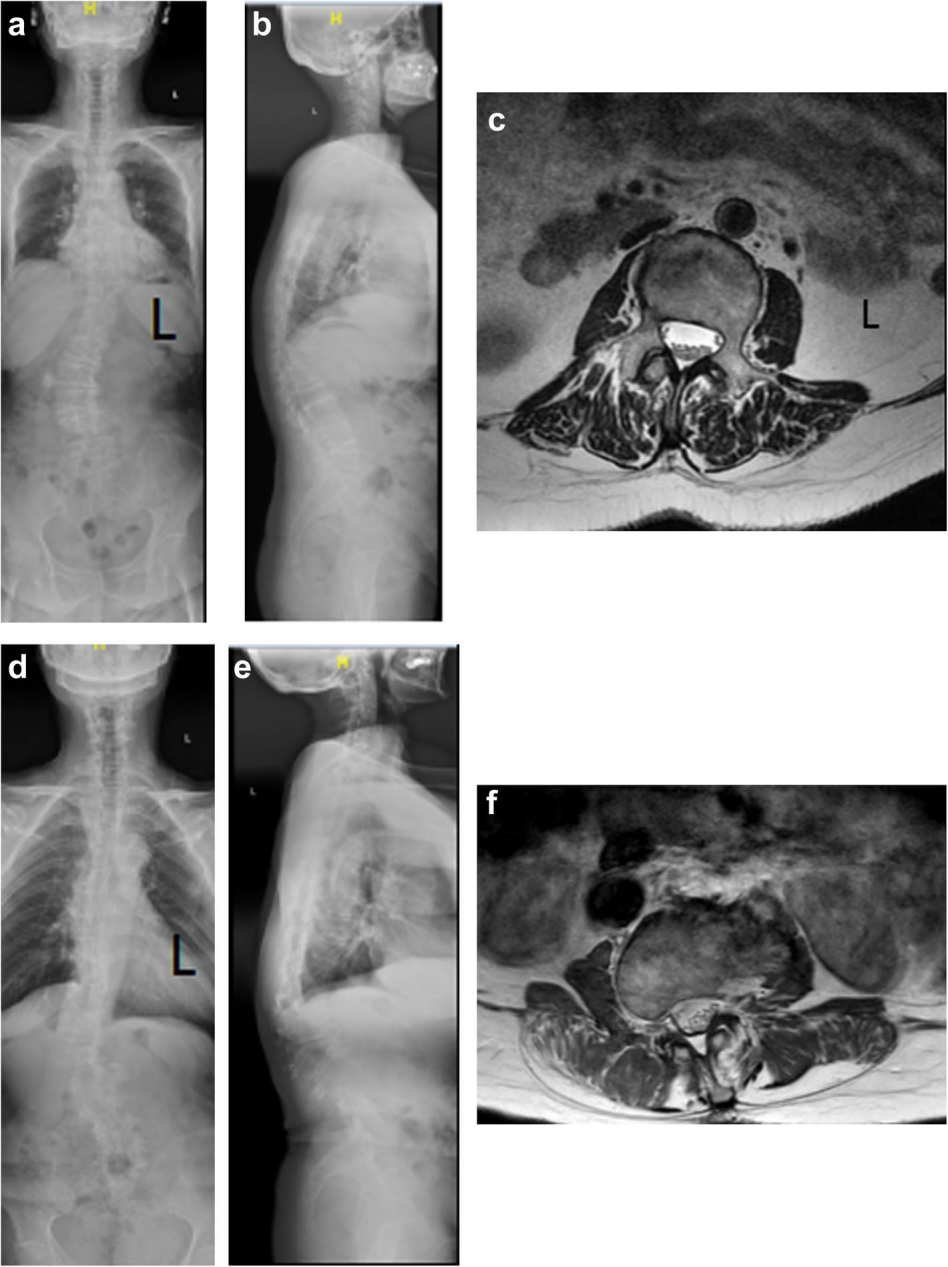
The standard standing whole spine posterior-anterior and lateral X-ray and lumbar spine MRI T2-weighted axis image of the RS and RKS groups. **a–c** The whole spine X-ray and L3 level MRI of 63-year-old women in the RS group. The TLK is 5.7° with no defined kyphosis. The apical vertebrae is L3 and Cobb angle is 22.2° with *α* of − 4.8°, *γ* of − 13.8°, and *d* of 4.73 cm. **d–f** The whole spine X-ray and L3 level MRI of 68-year-old women in the LKS group. The TLK is 43.1° with kyphosis. The apical vertebrae is L3 and Cobb angle is 23.3° with *α* of 6.6°, *γ* of − 16.0°, and *d* of 6.45 cm

There were no significant differences in *α* between the RS (− 5.74 ± 9.03°) and control groups (− 3.56 ± 6.40°) (*P* = 0.051), RKS (− 2.87 ± 10.79°) and control groups (*P* = 0.591), or RS and RKS groups (*P* = 0.072). There were significant differences in *d* when the RS (4.85 ± 0.46 cm) and RKS (5.11 ± 0.65) groups were compared with the control group (*P* < 0.001, *P* < 0.001, and *P* = 0.008, respectively) (Table 4, Fig. 3).

**Table 4:** Comparisons on *α* and *d* from T12 to L4 among the RS, RKS, and control groups

		RS vs control		RKS vs control		RS vs RKS	
*α*, °	T12	− 4.33 ± 12.73	− 1.94 ± 7.02	− 4.43 ± 15.31	− 1.94 ± 7.02	− 4.33 ± 12.73	− 4.43 ± 15.31
	L1	− 3.02 ± 9.35	0.30 ± 5.20	− 0.96 ± 6.84	0.30 ± 5.20	− 3.02 ± 9.35	− 0.96 ± 6.84
	L2	− 2.39 ± 4.59	− 3.17 ± 4.81	1.01 ± 9.64	− 3.17 ± 4.81	− 2.39 ± 4.59	1.01 ± 9.64
	L3	− 8.55 ± 8.31	− 5.41 ± 5.82	− 4.31 ± 14.17	− 5.41 ± 5.82	− 8.55 ± 8.31	− 4.31 ± 14.17
	L4	− 10.41 ± 6.98	− 7.63 ± 6.13	− 5.66 ± 6.93	− 7.63 ± 6.13	− 10.41 ± 6.98	− 5.66 ± 6.93
	Mean	− 5.54 ± 9.03	− 3.86 ± 6.40	− 2.87 ± 10.79	− 3.56 ± 6.40	− 5.74 ± 9.03	− 2.87 ± 10.79
*d*, cm	T12	4.41 ± 0.46**	4.02 ± 0.39	4.38 ± 0.56*	4.02 ± 0.39	4.41 ± 0.46	4.38 ± 0.56
	L1	4.66 ± 0.30**	4.26 ± 0.29	4.94 ± 0.46**	4.26 ± 0.29	4.66 ± 0.30	4.94 ± 0.46
	L2	4.90 ± 0.32**	4.50 ± 0.30	5.29 ± 0.50**	4.50 ± 0.30	4.90 ± 0.32*	5.29 ± 0.50
	L3	5.12 ± 0.40**	4.70 ± 0.34	5.57 ± 0.44**	4.70 ± 0.34	5.12 ± 0.40*	5.57 ± 0.44
	L4	5.15 ± 0.39**	4.71 ± 0.38	5.39 ± 0.59**	4.71 ± 0.38	5.15 ± 0.39	5.39 ± 0.59
	Mean	4.85 ± 0.46**	4.45 ± 0.43	5.11 ± 0.65**	4.45 ± 0.43	4.85 ± 0.46**	5.11 ± 0.65

*RS* right scoliosis group, *RKS* right kyphoscoliosis group

*Significance with *P* < 0.05

**Significance with *P* < 0.01

### Correlation analysis

The Pearson correlation analysis demonstrated a positive correlation between *d* and TLK (*r* = 0.439, *P* = 0.012), while *α* or *d* was not significantly correlated with Cobb angle (*P* > 0.05) and coronal horizontal displacement distance (*P* > 0.05). The results demonstrated that a severe thoracolumbar column kyphosis may lead to a farther distance away from the aorta (Table 5).

**Table 5:** Pearson correlation analysis between Cobb angle, the horizontal displacement, TLK, and *α* and *d*

	*α*		*d*	
	*r*	*P*	*r*	*P*
Cobb angle	0.019	0.899	0.052	0.733
Coronal movement	0.050	0.740	0.779	0.060
TLK	− 0.029	0.871	0.439	0.012

*TLK* thoracolumbar kyphosis

## Discussion

ADS usually resulted from asymmetric disc space collapse and facet degeneration with subsequent lateral and rotatory listhesis. Such degeneration commonly leads to a large curve in the lumbar spine and malalignment of the sagittal plane [12, 13]. Due to the lumbar curve, vertebrae rotatory, and lateral listhesis, the relative position of the aorta and the spine may be changed. Studies on the relative position of the aorta and the spine in AIS patients demonstrated that the position of the aorta changed at different curves [6–8, 14]. In patients with AS [9] and Pott’s diseases [10], the aorta is prone to move anteriorly and medially to the vertebrae. However, the relative position of the aorta in ADS patients remains unclear. Since ADS patients are usually accompanied by advanced age and reduced vascular elasticity, most patients suffered from atherosclerosis [15, 16]. Therefore, it is important to have a clear understanding on the anatomy of aorta to guide the physician in intraoperative manipulation and reduce the vascular-related complications.

The thoracic aorta begins at the aortic arch and runs anterior to the middle axis of the vertebral body. The position of the aorta changes with the arrangement of spine. According to the theory of tethering effect, the aorta is constraint by the surrounding crux in the diaphragm and forced into the concave side of the curves as it is the shortest distance between the top and the bottom of the chest cavity. The relative position of the aorta and the spine in AIS patients could be explained by this theory in multiple studies. Milbrandt and Sucato [6] proved that the thoracic aorta shifted to the left side of the curves and moved left laterally and posteriorly to the vertebral body in right thoracic curves in AIS patients. On the contrary, the thoracic aorta moved to the right and anterior to the vertebral body in left thoracic curves. Liljenqvist et al. and Sevastik et al. [17, 18] investigated the relative position of the aorta in AIS patients. It was found that the lateral displacement is larger but the vertical displacement is shorter in AIS patients.

However, the relative position of the aorta in ADS patients is totally different. In our study, 132 patients without spine deformity and 90 patients with ADS were recruited. There was no significant difference in the left pedicle-aorta angle between normal people and ADS patients. AIS is commonly featured as a regular and smooth curve with a larger Cobb angle, and the typical characteristic is the axis rotation contributing to three-dimensional malformation. The rotation of the vertebrate plays an important role in changing the relative position of the aorta in AIS patients. On the contrary, ADS is mainly caused by the degeneration of intervertebral discs, facets, and paravertebral muscles, in which the curve is irregular and the Cobb angle is usually less than 40°. In ADS, the typical characteristics are moderate rotation of the vertebrae and lateral listhesis, while the rotation is limited in the apical levels [19, 20]. Besides, most ADS patients have advanced age, the vascular elasticity is reduced, and the tethering ability of connective tissues is weakened, which is beneficial in maintaining a normal anatomical vertebrae-aorta position. Therefore, the degree of malformation is ameliorated and the relative position of the aorta is not obviously changed in ADS patients.

The left pedicle-aorta distance in ADS patients with kyphosis is increased compared with the control group and the group of ADS without kyphosis. The results indicated that kyphosis plays an important role in the changes of the distance, which was consistent with the previous studies [9, 10]. The Pearson correlation analysis demonstrated a positive correlation between the left pedicle-aorta distance and TLK, indicating that a severe thoracolumbar column kyphosis might lead to a farther distance between the spine and aorta. In the RS group, the distance between the aorta and the left pedicle was larger than that in the normal group, although no such trend was seen in the LS group. In the RS group, the vertebrae tended to rotate to the right and the left pedicle moved ventrally. The increase in the coronal displacement away from the aorta might overcompensate for the decrease in ventral movement towards the aorta and contribute to a longer left pedicle-aorta distance compared with the normal group.

In the spine surgeries, vascular injury is a rare but severe complication, which is manifested as acute bleeding, pseudoaneurysm, or arteriovenous fistula [4, 5]. Kulkarni et al. has specifically reported vascular complications following spine surgeries [5]. Liu indicated that the risk of aorta injury caused by the misplacement of screws would increase due to the vertebrae rotation and aliment change [21]. Various potential risk factors of aorta injury exist in ADS patients. Due to the fusion and osteophytes in normal structure, the anatomic position would change. ADS patients were commonly complicated by osteoporosis which may lead to the destruction of the anterior vertebrae. Besides, the insertion of pedicle screws was tricky and risky, and the misplaced screws in ADS patients would result in aorta injury. Moreover, in patients with severe or rigid ADS, the deformity correction surgery was necessary and sometimes osteotomy was performed to restore a good balance. Previous reports have proved that aorta injury would occur in osteotomy surgery [22, 23]. Furthermore, ADS patients are usually in an advanced age, so the vascular elasticity is reduced and commonly complicated by atherosclerosis. Ayca et al. [24] reported the correlation between ADS and the diameter of aorta, and it was found that the aorta in ADS patients was more vulnerable to be injured. However, the relative position of the aorta and the spine in ADS patients still remains unclear, which is of great importance for further research.

There are some limitations in this study. First, patients usually take supine position during MR examinations, but the surgery is usually performed in prone position. Whether the position of the aorta changes from supine to prone position is not clear. Second, kyphosis deformities in ADS patients are not always located in thoracolumbar regions but sometimes in lumbar column; thus, the conclusion may not be applicable for all ADS cases. Last, the sample number in our study is relatively small, so more cases are needed in further research.

## Conclusion

In conclusion, the left pedicle-aorta angle maintained relatively normal in ADS patients. The aorta shifted slightly away from the left pedicle in ADS patients with RS and kyphosis, especially in those with kyphosis. However, the shift was not obvious in ADS patients with LS. Moreover, the aorta-vertebrae distance was significantly correlated with TLK. The surgeon should evaluate the position of the aorta before operations to avoid vessel injuries. Besides, the vertebral rotation angle is an important parameter to evaluate the severity of the deformity in ADS patients.

## Data Availability

Please contact the author for data requests.

## Abbreviations

ADS: Adult degenerative scoliosis
LKS: Left scoliosis with thoracolumbar kyphosis
LS: Left scoliosis
RKS: Right scoliosis with thoracolumbar kyphosis
RS: Right scoliosis
TLK: The sagittal angle between superior endplate of T10 and inferior endplate of L2
